# Mental Defeat Predicts Increased Suicide Risk in Chronic Pain: A 12‐Month Prospective Study

**DOI:** 10.1002/ejp.4779

**Published:** 2025-01-14

**Authors:** Kristy Themelis, Jenna L. Gillett, Paige Karadag, Martin D. Cheatle, Mark A. Ilgen, Shyam Balasubramanian, Swaran P. Singh, Nicole K. Y. Tang

**Affiliations:** ^1^ Department of Psychology University of Warwick Coventry UK; ^2^ Department of Psychology Nottingham Trent University Nottingham UK; ^3^ Department of Psychology University of Buckingham Buckingham UK; ^4^ Department of Psychiatry and Anesthesiology and Critical Care Perelman School of Medicine University of Pennsylvania Philadelphia Pennsylvania USA; ^5^ Department of Veteran Affairs, Center for Clinical Management Research VA Ann Arbor Healthcare System Ann Arbor Michigan USA; ^6^ Department of Psychiatry University of Michigan Medical School Ann Arbor Michigan USA; ^7^ UHCW NHS Trust Coventry UK; ^8^ Warwick Medical School Coventry UK

## Abstract

**Background:**

Mental defeat is considered a potential risk factor for suicidal thoughts and behaviours in chronic pain. This study evaluated the role of mental defeat in predicting future suicide risk and examined whether depression influences this relationship.

**Methods:**

A total of 340 participants with chronic pain completed questionnaires at two time points, 12 months apart. Data collected included sociodemographic and pain characteristics, mental defeat, psychosocial risk factors including depression and health‐related variables. Weighted univariate and multivariable analyses assessed the link between mental defeat and suicide risk, with a moderation analysis testing the role of depression.

**Results:**

Higher levels of mental defeat and depression were linked to increased suicide risk at 12 months. Depression significantly amplified the effect of mental defeat on suicide risk, particularly in individuals with higher depression levels (*B* = 0.06, SE = 0.01, *t* = 6.21, *p* < 0.001) compared with moderate (*B* = 0.05, SE = 0.01, *t* = 5.20, *p* < 0.001) or low levels of depression (*B* = 0.04, SE = 0.01, *t* = 2.83, *p* = 0.004), indicating a dose–response relationship.

**Conclusions:**

Mental defeat is a significant risk factor for suicide in chronic pain, with depression intensifying this risk. Addressing both mental defeat and depression simultaneously in treatment may help reduce suicide risk in these patients.

**Significance:**

This study strengthens the evidence linking mental defeat with heightened suicide risk in chronic pain. By providing prospective data, it clarifies the temporality of this relationship. Given that suicide risk doubles in chronic pain patients, whereby comorbid depression is common, these findings have crucial clinical implications. Both mental defeat and depression are modifiable. Addressing them together in treatment may help reduce suicide risk in this population.

## 1 Introduction

Living with chronic pain significantly affects daily life and carries substantial financial and mental health burdens (Dueñas et al. 2016; Fayaz et al. 2016; Goldberg and McGee 2011). The connection between chronic pain and mental health issues, particularly suicide, is becoming more widely recognised. Individuals with chronic pain are at double the risk of suicide compared to the general population (Campbell et al. 2015; Cheatle et al. 2014; Tang and Crane 2006). Chronic pain was present in approximately 1 in 10 suicide cases, and over half of those individuals mentioned pain as a factor in their decision to die by suicide (Petrosky et al. 2018). Despite the known progression from suicidal ideation and non‐fatal suicidal behaviour to death by suicide, these warning signs are often overlooked (Costanza et al. 2021; Fairweather‐Schmidt et al. 2016).

Research has identified various general and pain‐specific risk factors for suicidal thoughts and behaviour in people with chronic pain. General risk factors include unemployment, reliance on disability benefits, childhood adversity, exposure to domestic violence, family history of depression or suicidal behaviour, depressive symptoms and substance use. Pain‐specific risk factors include the type and number of pain conditions, frequent or intermittent pain, sleep disturbances and high levels of pain catastrophizing. Two systematic reviews have underscored the role of psychosocial factors—pain catastrophizing, mental defeat, feelings of burdensomeness and thwarted belongingness—as key drivers of suicide risk in individuals with chronic pain (Racine 2018; Tang and Crane 2006). Mental defeat has emerged as a modifiable risk factor for suicide prevention (Racine 2018). Originating from trauma and depression research (Ehlers et al. 1998; Gilbert and Allan 1998), mental defeat refers to negative self‐appraisals related to pain, stemming from perceived losses of autonomy, agency, social status and integrity. Repetitive experiences of persistent pain are believed to be recurrent triggers for the experience of defeat. Mental defeat, as measured with the Pain Self Perception Scale (Tang, Salkovskis, and Hanna 2007), offers a direct means to assess pain‐related suffering especially in relation to chronic pain's impact on self‐identity. Those seeking specialised treatment for chronic pain often exhibit higher levels of mental defeat, distress and disability (Tang et al. 2010). Mental defeat has also been consistently linked to suicidal thoughts and behaviours in cross‐sectional studies (Cheatle et al. 2023; Tang, Beckwith, and Ashworth 2016).

Our prior research identified mental defeat as a prospective risk factor for suicide in individuals with chronic pain, alongside depression, perceived stress, headaches and smoking (Themelis et al. 2023). However, the long‐term relationship between mental defeat and suicide risk, and the potential moderating role of depression, is less understood. Many individuals with chronic pain experience moderate to severe depression or have comorbid depressive disorders, which may further elevate their risk of suicide (Bair et al. 2003). The literature suggests that mental defeat may amplify suicide risk in individuals with mental health conditions like depression or schizophrenia (Johnson, Gooding, and Tarrier 2008; Tang, Salkovskis, and Hanna 2007).

In this study, we followed individuals with chronic pain over 12 months, assessing them at two points to determine whether (1) mental defeat predicts suicide risk after 1 year and (2) depression moderates the relationship between mental defeat and suicide risk.

## 2 Method

### 2.1 Study Design and Sample

This study was a prospective cohort design that formed part of the wider Warwick Study of Mental Defeat in Chronic Pain (‘WITHIN’ study). Participants based in the United Kingdom were recruited between March 2020 and August 2021 via opportunity sampling through various platforms, including pain‐related charities, social media, recruitment sites, patient support groups, pain clinics, physiotherapists and local public engagement events. Potential participants were screened for eligibility and had to be (1) aged between 18 and 65 years at the time of enrolment, (2) experience chronic non‐cancer pain (CNCP) for at least 3 months, (3) be on a stable treatment regimen and (4) be able to provide informed consent. Exclusion criteria included: (1) self‐reported presence of severe comorbid medical, neurological or psychiatric conditions, (2) scheduled elective surgery or procedures requiring general anaesthesia during the study and (3) participation in another research study involving an investigational product within the last 12 weeks. This study was approved by the West Midlands‐Solihull Research Ethics Committee on 26/06/2017 (reference: 17/WM/0053, IRAS project ID: 223190) and electronic informed consent was obtained from all participants.

This study originally collected questionnaire data at three different time points (baseline, 6 months and 12 months). Results from the 6‐month mark have already been published (Themelis et al. 2023). The present manuscript focuses on assessing longer‐term prospective relationships and thus utilises the baseline and 12‐month assessment points of the wider study to explore the psychosocial predictors of suicide risk in chronic pain and the potential role of depression in moderating the mental defeat‐suicide risk relationship. Out of the initial sample (*n* = 524) that completed baseline measures, *n* = 340 (64.8%) completed the follow‐up assessment at 12 months and provided information on the measure of suicide risk (SBQ‐R).

### 2.2 Instruments

#### 2.2.1 Screening Variables and Medical History

A sociodemographic screening questionnaire was implemented to indicate age, gender, ethnicity, education level (tertiary/secondary or below), employment status (working /not working), pain aetiology and medical history. The screening and main questionnaires were administered using Qualtrics software (Qualtrics, Provo, UT). To assess health and pain‐related characteristics, the screening survey also included questions on smoking status, current alcohol habits, pain location (via a hot‐spot body map), pain duration (up to a maximum of 30+ years), any treatment plans and medication use. The presence of widespread pain was determined based on self‐reported pain locations, in line with the definition by the International Association for the Study of Pain (IASP) (Treede et al. 2019). The Medication Quantification Scale‐III (MQS‐II) (Harden et al. 2005), was used to quantify medication use into a single score. This validated scoring system was adapted by NHS‐registered clinicians on the study team to reflect current prescription practices in the United Kingdom.

#### 2.2.2 Questionnaire

The questionnaire measures included measurements of suicide risk as the primary outcome and associated psychosocial predictors. Predictors of suicide risk included psychological states (mental defeat, pain catastrophizing, pain‐related efficacy, anxiety, depression, perceived stress, pain vigilance and fear of movement) and pain and health characteristics (pain location, the presence of widespread pain, pain severity, pain interference and insomnia severity), all of which were hypothesised risk factors for suicidal thoughts and behaviours informed by prior research (Themelis et al. 2023). The primary outcome of suicide risk (SBQ‐R), the key predictor of interest (mental defeat) and the moderator (depression) are briefly described below (Table 1 for a full list of questionnaire measures). Internal consistency was calculated and reported for each measure consisting of multiple questions in this sample using Cronbach's alpha (*α*).

**TABLE 1:** Validated questionnaire measures included.

Measure	Scale used	Example statements	Scoring
Suicide risk			
Suicidal behaviour	Suicidal Behaviour Questionnaire‐Revised (SBQ‐R) (Osman et al. 2001)	‘How likely is it that you will attempt suicide someday?’	The total score ranges from 3 to 18. A cutoff of ≥ 7 was used to dichotomise participants into low‐risk and high‐risk
Psychological states			
Mental defeat	Pain Self‐Perception Scale (PSPS) (Tang, Salkovskis, and Hanna 2007)	‘Because of the pain—I felt that I had sunk to the bottom of the ladder’	A total score ranging from 0 to 96 where a higher score indicates a heightened sense of mental defeat
Pain catastrophizing	Pain Catastrophizing Scale (PCS) (Sullivan, Bishop, and Pivik 1995)	‘I become afraid that the pain will get worse’	A total score ranging from 0 to 52, where higher scores indicate higher levels of pain catastrophizing
Pain self‐efficacy	Pain Self‐Efficacy Questionnaire (PSEQ) (Nicholas 2007)	‘I can enjoy things, despite the pain’	A total score ranging from 0 to 60. A higher score indicates higher pain self‐efficacy
Anxiety	HADS‐A—Hospital Anxiety & Depression Scale (HADS) (Zigmond and Snaith 1983)	‘I get a sort of a frightened feeling like “butterflies”’	Scores ranging from 0 to 21, with a higher score indicating worsened symptoms
Depression	HADS‐D‐ Hospital Anxiety & Depression Scale (HADS) (Zigmond and Snaith 1983)	‘I still enjoy the things I used to enjoy’	Scores ranging from 0 to 21, with a higher score indicating worsened symptoms
Perceived stress	Perceived Stress Scale (PSS) (Cohen, Kamarck, and Mermelstein 1983)	‘In the last month, how often have you been upset because of something that happened unexpectedly?’	A total stress score is calculated by summing all items with scores ranging from 0 to 40. A higher score indicates more stress
Pain vigilance	Pain Vigilance and Awareness Questionnaire (PVAQ) (Roelofs et al. 2003)	‘I am very sensitive to pain’	The total score ranges from 0 to 80 where a higher score indicates higher pain vigilance and awareness
Fear of movement	Tampa Scale of Kinesiophobia (TSK‐11) (Woby et al. 2005)	‘I'm afraid that I might injure myself if I exercise’	The total fear of painful movement and injury score is calculated by summing all the items with scores ranging from 11 to 44. A higher score indicates being more fearful
Pain‐related measures			
Pain severity	BPI Severity—Brief Pain Inventory‐Short Form (BPI‐SF) (Cleeland and Ryan 1994)	‘Please rate your pain by circling the one number that best describes your pain at its worst in the last week’.	Averaging four severity items ranging from 0 to 10. Higher scores indicate greater severity.
Pain interference	BPI Interference‐ Brief Pain Inventory‐Short Form (BPI‐SF) (Cleeland and Ryan 1994)	‘Circle the one number that describes how, during the past week, pain has interfered with your general activity’.	Averaging seven interference items ranging from 0 to 10. Higher scores indicate more interference.
Body Map Index	Body Pain Map (Cleeland and Ryan 1994)	‘Which area hurts the most?’	A Body‐map index was calculated by adding up the total amount of selected body areas out of 42.
Activity and health variables			
Insomnia symptom severity	Insomnia Severity Index (ISI) (Bastien, Vallières, and Morin 2001)	‘How satisfied/dissatisfied are you with your current sleep pattern?’	The total summed score ranges from 0 to 28 where higher scores indicate more severe insomnia. The score can be interpreted into 4 categories with the absence of insomnia (0–7), mild (8–14), moderate (15–21) and severe insomnia (22–28)
Physical activity	International Physical Activity Questionnaire (IPAQ) (Craig et al. 2003)	‘During the last 7 days, on how many days did you do vigorous physical activities like heavy lifting, digging, aerobics, or fast bicycling?’	MET values were calculated using a spreadsheet made freely available online (Cheng 2016). We report the frequency of participants in the IPAQ low‐activity group as a demographic variable
Social activity	Social Activity Log (SAL) (Syrjala et al. 2010)	‘How many days in the past week did you do voluntary social activities?’	The total score ranged from 0 to 91 where higher scores indicated more social activity
Patterns of activity (pain‐specific)	Patterns of Activity Measure for Pain (POAM‐P) (Cane et al. 2013)	‘When I'm doing an activity, I don't stop until it is finished’	The total score for each subscale ranges from 0 to 50 (150 in total), where a higher score indicates more avoidance, pacing or overdoing, respectively

#### 2.2.3 Primary Outcome: Suicide Risk

Suicide risk was assessed using the 4‐item Suicidal Behaviours Questionnaire‐Revised (SBQ‐R) (Osman et al. 2001). This questionnaire evaluates lifetime suicide ideation, frequency of ideation within the past 12 months, the threat of suicidal behaviour and self‐reported likelihood of suicidal behaviour. The total score ranges from 3 to 18, with a score of ≥ 7 indicating a high risk of suicidal behaviour in the general population, and a score of ≥ 8 indicating a high risk in clinical or inpatient populations. In accordance with previous research (Themelis et al. 2023), we used the cut‐off of ≥ 7 to categorise participants into low‐risk and high‐risk groups. The SBQ‐R score was chosen due to its high test‐score reliability, sensitivity (93%) and specificity (95%), as well as its brevity, ease of administration and widespread use in both clinical practice and research (Osman et al. 2001). As a further safety and ethical precaution, participants who scored above clinical cut‐off (≥ 7) during any assessment point were followed up by a member of the research team via email and/or telephone call (if consented to by the participant) with signposting to relevant support services.

#### 2.2.4 Key Predictor of Interest: Mental Defeat

The key predictor of interest was mental defeat, measured by the 24‐item Pain Self‐Perception Scale (PSPS) (Tang, Salkovskis, and Hanna 2007). Six example statements within the measure with the highest item‐total correlations in the validation study are: ‘Because of the pain…I felt that I had sunk to the bottom of the ladder’; ‘…I felt destroyed as a person’; ‘…I felt that life had treated me like a punchbag’; ‘…I felt defeated’; ‘…I felt that my confidence had been knocked out of me’; ‘…in my mind, I gave up’. Items are scored on a 0–4 Likert scale, where 0= ‘Not at all/Never’ and 4 = ‘Very strongly’. A total score is generated ranging from 0 to 96, with a higher score indicating a heightened sense of mental defeat.

#### 2.2.5 Moderator: Depression

The moderator of interest was depression measured using the 14‐item Hospital Anxiety and Depression Scale (HADS) (Zigmond and Snaith 1983). Example statements within the measure is: ‘I still enjoy the things I used to enjoy’; ‘I feel cheerful’; ‘I feel as if I am slowed down’ and ‘I have lost interest in my appearance’. Items are scored on a 4‐point Likert scale, with different anchors for each item. There are seven items measuring depression (HADS‐D) with scores ranging from 0 to 21, with a higher score indicating worsened symptoms.

### 2.3 Data Analysis

Data analyses were conducted using IBM SPSS statistics software (version 29.01) and R Studio (version 4.3.2 for Mac). To account for attrition bias, both the univariate and multivariable models were weighted for age, sex, ethnicity, education level, employment status, alcohol consumption, smoking status and pain medication using inverse probability weighting (Seaman and White 2013; Thoemmes and Ong 2016). Complete case analysis was used, and we did not rely on data imputation.

First, a univariate regression model was constructed to examine the association between each baseline demographic and clinical characteristic and the odds ratio of participants reporting a SBQ‐R score of ≥ 7 at 12 months independently. Following this, a multivariable model was constructed by adding only factors that were observed to be significant predictors (*p* < 0.05) in the logistic regression model with the SBQ‐R score at 12 months as the dependent variable. Variance Inflation Factors (VIF) were calculated to check for potential multicollinearity. Variables with a VIF greater than 4 were excluded in the final model.

Moderation analyses were conducted using the PROCESS function for R/RStudio (Hayes 2021) to explore depression as a potential moderator of mental defeat in predicting 12‐month suicide risk. This function uses bootstrapped regression to test the moderation effects within models. The number of bootstraps for percentile bootstrap confidence intervals that were used was 10,000. The mental defeat and depression variables were mean‐centred before analysis and the depression and mental defeat interaction term was computed (Aiken and West 1991). The two predictors and the interaction were entered into a simultaneous regression model. Significant interaction effects were followed up using a simple slope analysis with low (1 SD below the mean), moderate (mean) and high (1 SD above the mean) levels of depression. The analysis was considered significant at *p* < 0.05. Unstandardised beta values are reported.

## 3 Results

### 3.1 Study Overview

The group of participants (*n* = 340) at baseline, who subsequently completed the follow‐up assessment, was primarily white (90%) and female (83%) with an average age of 40 years (SD 12.28). A significant proportion (89%) of participants reported having educational qualifications beyond secondary school. Over one‐third (42%) of participants reported not being currently employed. Only 17% (17%) of participants were active smokers/vapers and 12% reported frequent alcohol use more than three times a week. In this research study, 38.5% met the cut‐off score for high risk for suicide at baseline (SBQ‐R score: mean = 6.36, SD = 3.64); 37.9% at 12 months (mean = 6.25, SD = 3.75). More information on the participant characteristics for the total sample and subsamples at baseline can be found in Table 2.

**TABLE 2:** Participant characteristics for the total sample (*N* = 524) and for subsamples at baseline.

			Subsamples				
	Total (*n* = 524)	*α*	Low risk (*n* = 319)^a^	High risk (*n* = 202)^a^	*x* ^2^ */t*	*p*	*d/ϕc*
Sociodemographics							
Age^b^	39.9 (12.3)		40.6 (12.4)	38.9 (12.2)	1.53	0.13	0.14
Sex, *n* (%)					11.54	**< 0.001**	0.15
Female	420 (80.2)		267 (82.9)	153 (75.7)			
Male	98 (18.7)		55 (17.1)	43 (21.3)			
Other	6 (1.2)		0 (0.0)	6 (3.0)			
Ethnicity, *n* (%)					4.39	0.36	0.09
White	471 (89.9)		291 (90.4)	180 (89.1)			
Asian/Asian British	23 (4.4)		15 (4.7)	8 (4.0)			
Mixed/Multiple ethnic groups	17 (3.2)		10 (3.1)	7 (3.5)			
Black/African/Caribbean/Black British	11 (2.1)		4 (1.2)	7 (3.5)			
Prefer not to say	2 (0.4)		2 (0.6)	0 (0.0)			
BMI^b^	28.7 (7.8)		28.5 (7.4)	28.9 (8.4)	−0.53	0.60	−0.05
BMI category^b^, *n* (%)					0.03	0.87	0.01
BMI < 25	197 (37.6)		120 (37.3)	77 (38.1)			
BMI ≥ 25	326 (62.2)		201 (62.6)	125 (61.9)			
Education, *n* (%)					0.10	0.75	0.01
Secondary or below	60 (11.5)		38 (11.8)	22 (10.9)			
Tertiary	464 (88.6)		284 (88.2)	180 (89.1)			
Not working, *n* (%)	208 (39.7)		108 (33.5)	100 (49.5)	13.22	**< 0.001**	0.16
Active smoker/vaper, *n* (%)	94 (17.9)		40 (12.4)	54 (26.7)	17.27	**< 0.001**	0.18
Frequent alcohol consumption, *n* (%)	72 (13.7)		44 (13.7)	28 (13.9)	0.01	0.95	0.01
Total MQS score^b^	3.8 (6.5)		3.7 (6.8)	3.9 (6.2)	−0.35	0.73	−0.03
Suicide risk							
SBQ‐R	6.4 (3.6)	0.83	3.9 (1.1)	10.3 (2.7)	−38.09	**< 0.001**	−3.42
Psychological states							
Mental defeat (PSPS)	35.5 (25.0)	0.98	28.0 (21.4)	47.5 (25.7)	−9.37	**< 0.001**	−0.84
Depression (HADS‐D)	7.9 (4.3)	0.81	6.6 (3.9)	9.9 (4.5)	−9.10	**< 0.001**	−0.82
Anxiety (HADS‐A)	9.4 (4.7)	0.85	7.81 (4.6)	11.4 (4.4)	−8.44	**< 0.001**	−0.76
Pain catastrophizing (PCS)	22.9 (12.4)	0.95	20.0 (11.3)	27.4 (12.7)	−6.93	**< 0.001**	−0.62
Pain self‐efficacy (PSEQ)	33.4 (14.3)	0.94	36.5 (13.3)	28.4 (14.4)	6.56	**< 0.001**	0.59
Perceived stress (PSS)	21.7 (7.2)	0.88	19.6 (6.7)	25.1 (6.5)	−9.17	**< 0.001**	−0.82
Pain vigilance (PVAQ)	48.1 (13.0)	0.88	47.4 (12.6)	49.3 (13.5)	−1.63	0.10	−0.15
Fear of movement (TSK)	26.9 (6.7)	0.85	25.9 (6.2)	28.6 (7.0)	−4.54	**< 0.001**	−0.41
Pain‐related measures							
Pain duration (years)	10.0 (8.3)		9.7 (8.2)	10.3 (8.6)	−0.79	0.43	−0.07
Pain location, *n* (%)							
Head	167 (31.9)		88 (27.3)	79 (39.1)	7.93	**< 0.001**	0.12
Back	338 (64.5)		193 (59.9)	145 (71.8)	7.61	**< 0.001**	0.12
Shoulder/arm	300 (57.3)		177 (55.0)	123 (60.9)	1.78	0.18	0.06
Trunk	271 (51.7)		152 (47.2)	119 (58.9)	6.81	**< 0.001**	0.11
Hip/buttocks/legs	356 (67.9)		217 (67.4)	139 (68.8)	0.12	0.73	0.01
> 1 pain location, *n* (%)	391 (74.6)		231 (71.7)	160 (79.2)	3.66	0.06	0.08
Widespread pain, *n* (%)	190 (36.3)		103 (32.0)	87 (43.1)	6.46	**0.01**	0.11
Pain severity (BPI severity)	4.6 (1.8)	0.88	4.4 (1.8)	5.1 (1.7)	−4.34	**< 0.001**	−0.39
Pain interference (BPI interference)	5.3 (2.5)	0.93	4.9 (2.6)	6.0 (2.3)	−4.91	**< 0.001**	−0.44
Body Map Index	10.9 (10.4)	0.95	9.9 (9.8)	12.4 (11.2)	−2.65	**< 0.001**	−0.24
Activity and health variables							
Insomnia severity (ISI)	13.0 (7.7)	0.93	11.9 (7.6)	14.7 (7.7)	−4.13	**< 0.001**	−0.37
Low physical activity (IPAQ), *n* (%)	196 (37.4)		113 (35.1)	83 (41.1)	1.91	0.17	0.06
Social activity (SAL)	15.3 (9.1)	0.67	15.7 (8.5)	14.8 (10.0)	1.14	0.25	0.10
Patterns of activity measure (POAM‐P)							
Avoidance	22.5 (8.5)	0.89	21.8 (8.3)	23.7 (8.8)	−2.51	0.01	−0.22
Overdoing	24.4 (7.0)	0.83	24.4 (6.9)	24.4 (7.1)	0.01	0.99	0.01
Pacing	21.2 (9.1)	0.94	21.1 (8.8)	21.5 (9.4)	−0.46	0.65	−0.04

*Note:* Means are presented followed by standard deviations in parentheses unless otherwise specified. Internal consistency was calculated for each measure consisting of multiple questions in this sample at baseline using Cronbach's alpha (*α*). *T*‐tests for continuous variables and Chi‐square tests for categorical variables were used to test if SBQ‐R no/lower risk versus higher risk differs in demographics and pain characteristics at baseline. Continuous variables' effect size was computed using Cohen's *d* (*d*) and categorical variables' effect size was computed using Cramer *ϕ* (*ϕc*). The bold value indicates a significant difference between the low risk and high risk groups at baseline (*p* < 0.01). The following variables had between 1 and 4 missing values: Age (*n* = 522), BMI (*n* = 520), Total MQS score (*n* = 523), Mental defeat (*n* = 523), Pain self‐efficacy (*n* = 523), Pain vigilance (*n* = 523) and Fear of movement (*n* = 523).

Abbreviations: BMI, Body Mass Index; Frequent Alcohol consumption, defined as 3 or more units per week; MQS, The Medication Quantification Scale; Pain duration was measured in years at screening up to a maximum of 30+ years Body Map Index, the number of areas in which pain is present (42 areas in total); Tertiary, the educational level following the completion of secondary education; Not working, includes students and (medically) retired; Widespread pain, defined as pain present above and below the waist, in the right‐ and left‐hand sides of the body and in the axial skeleton (as per IASP and American College of Rheumatology 1990 definitions).

^a^ Low risk defined as a 0‐6 SBQ‐R score; High risk defined as ≥ 7 SBQ‐R score.

### 3.2 Predicting 12‐Month Suicide Risk

A univariate logistic model (*n* = 340) demonstrated that not working, elevated levels of mental defeat, pain catastrophizing, anxiety, depression, perceived stress, pain vigilance, fear of movement, pain severity, pain interference, insomnia severity and experiencing head pain, experiencing widespread pain, were all significant predictors of suicide risk at 12 months (*p* < 0.01). Higher pain self‐efficacy was found to reduce the risk (see Table 3).

**TABLE 3:** Results of univariate and multivariable analysis of predictors of suicide risk (SBQ‐R ≥ 7) at 12 months.

	Univariate analysis				Multivariable analysis			
	Odds ratio	95% CI		*p*	Odds ratio	95% CI		*p*
Sociodemographics								
Age	0.99	0.97	1.01	0.27	—	—	—	—
Male	1.45	0.81	2.58	0.21	—	—	—	—
Non‐white	1.94	0.95	3.96	0.07	—	—	—	—
BMI ≥ 25 (vs. 25 or lower)	1.35	0.86	2.14	0.19	—	—	—	—
Secondary education or below	0.75	0.36	1.59	0.46	—	—	—	—
Not working	**1.85**	**1.18**	**2.90**	**0.01**	1.53	0.90	2.62	0.12
Active smoker/vaper	1.72	0.97	3.04	0.06	—	—	—	—
Frequent alcohol consumption	0.82	0.42	1.60	0.56	—	—	—	—
Psychological states								
Mental defeat (PSPS)	**1.04**	**1.03**	**1.05**	**< 0.001**	**1.03**	1.01	1.05	**0.004**
Depression (HADS‐D)	**1.23**	**1.16**	**1.31**	**< 0.001**	**1.13**	1.03	1.24	**0.01**
Anxiety (HADS‐A)	**1.17**	**1.11**	**1.24**	**< 0.001**	**±±**	**±±**	±±	**±±**
Catastrophizing (PCS)	**1.07**	**1.04**	**1.09**	**< 0.001**	1.01	0.98	1.05	0.48
Pain self‐efficacy (PSEQ)	**0.96**	**0.94**	**0.97**	**< 0.001**	1.01	0.98	1.04	0.55
Perceived stress (PSS)	**1.13**	**1.09**	**1.17**	**< 0.001**	1.05	0.99	1.10	0.09
Pain Vigilance (PVAQ)	**1.02**	**1.00**	**1.04**	**0.03**	0.97	0.95	1.00	0.05
Fear of movement (TSK)	**1.08**	**1.04**	**1.12**	**< 0.001**	1.02	0.97	1.07	0.50
Pain‐related measures								
Pain duration (years)	1.02	0.99	1.05	0.20	—	—	—	—
Pain location (yes vs. no)					—	—	—	—
Head	**1.74**	**1.09**	**2.80**	**0.02**	1.73	0.94	3.18	0.08
Back	1.43	0.88	2.30	0.15	—	—	—	—
Shoulder/arm	1.39	0.88	2.18	0.16	—	—	—	—
Trunk	1.52	0.97	2.38	0.07	—	—	—	—
Hip/buttocks/legs	1.18	0.73	1.92	0.50	—	—	—	—
> 1 pain location	1.40	0.82	2.39	0.21	—	—	—	—
Widespread pain (yes vs. no)	**1.69**	**1.07**	**2.66**	**0.03**	1.27	0.69	2.35	0.44
Pain severity (BPI severity)	**1.21**	**1.07**	**1.37**	**0.01**	0.99	0.80	1.24	0.96
Pain interference (BPI interference)	**1.23**	**1.12**	**1.35**	**< 0.001**	0.97	0.80	1.18	0.76
Body Map Index	1.02	0.99	1.04	0.15	—	—	—	—
Activity and health variables								
Insomnia severity (ISI)	**1.05**	**1.02**	**1.08**	**< 0.001**	0.98	0.94	1.02	0.33
Low physical activity (IPAQ)	1.31	0.83	2.06	0.25	—	—	—	—
Social activity (SAL)	0.98	0.96	1.01	0.16	—	—	—	—
Patterns of activity measure (POAM‐P)								
Avoidance	1.02	1.00	1.05	0.10	—	—	—	—
Overdoing	1.01	0.98	1.05	0.39	—	—	—	—
Pacing	1.00	0.98	1.03	0.91	—	—	—	—

*Note:* Frequent alcohol consumption, defined as 3 or more units of alcohol per week. Multivariable model, Akaike information criteria (AIC): 378.092; Bayesian information criteria (BIC): 431.504; Likelihood‐ratio chi‐square: 90.817. The bold value indicates a *p*‐value = < 0.001. 95% CI, 95% confidence interval of odds ratio scale. ± HADS anxiety not included in the multivariable model due to multicollinearity with depression and a VIF > 4. –, Not included in the multivariable model as not significant in the univariate model.

A multivariable model (*n* = 340), including only significant univariate predictors, showed that elevated mental defeat (OR = 1.03, 95% CI: 1.01, 1.05, *p* = 0.004) and elevated depression (OR = 1.13, 95% CI: 1.03, 1.24, *p* = 0.01) were the only two variables that remained significant at 12‐month follow‐up (Table 3). Each 1‐point increase in mental defeat (PSPS; range: 0‐96) scores was associated with a 3% increase in the OR for suicide risk. For depression, each 1‐point increase in HADS‐D scores (range: 0‐21) was associated with a 13% increase in the OR for suicide risk. The Akaike information criterion (AIC) is 378.1 and the Bayesian information criteria (BIC) is 431.5. HADS anxiety was not included in the multivariable model due to multicollinearity with depression and a VIF > 4.

### 3.3 Assessing the Interaction Between Mental Defeat and Depression

**FIGURE 1:**
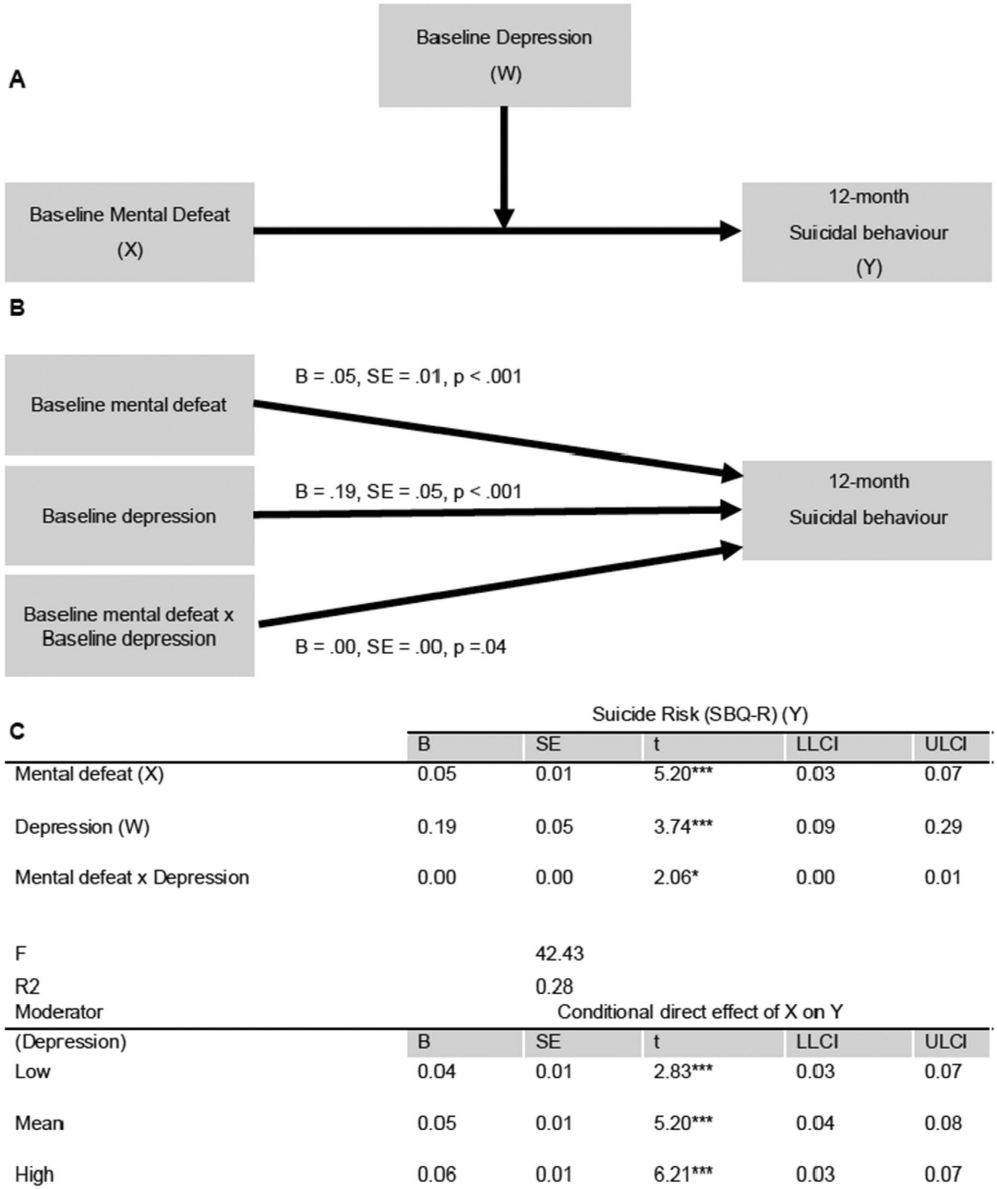
Moderation to explore depression as a potential moderator of mental defeat in predicting 12‐month suicide risk. (A) Conceptual model and (B) statistical model. Unstandardised beta values were reported. (C) Results of moderation analysis using PROCESS. *N* = 340. LLCI, lower limit confidence interval; UCLI, upper limit confidence interval. The bias‐corrected and accelerated 90% confidence intervals (CIs) are reported and calculated using 10,000 bootstrap samples. *p < 0.05. **p < 0.01. ***p < 0.001.

A moderation model was used to investigate the interaction between mental defeat and depression in predicting the risk of suicide at 12 months depending on levels of depression (Figure 1). The results of the moderation analysis using PROCESS are shown in Figure 1C. As illustrated in Figure 1B, greater levels of mental defeat (*B* = 0.05, SE = 0.01, *t* = 5.20, *p* < 0.001) and greater levels of depression (*B* = 0.19, SE = 0.05, *t* = 3.74, *p* < 0.001) were both independently associated with a higher risk of suicide at 12 months. The interaction between mental defeat and depression was also significant (*B* = 0.003, SE = 0.001, *t* = 2.06, *p* = 0.04), suggesting that the effect of mental defeat on the risk of suicide at 12 months depended on the level of depression. Together, the variables accounted for approximately 28% of the variance in risk of suicide, *R* ^2^ = 0.28, F(3,336) = 42.43, *p* < 0.001.

**FIGURE 2:**
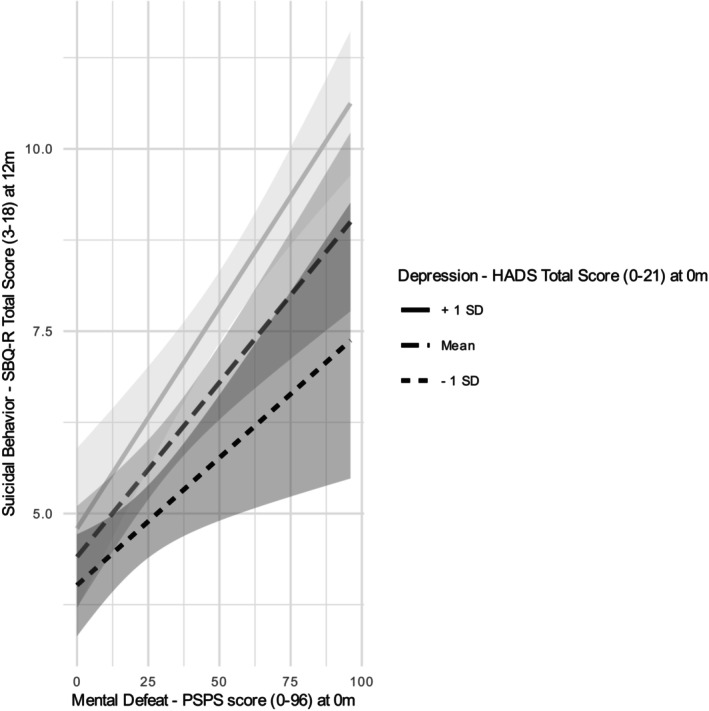
Simple slopes of the relationship between mental defeat and risk of suicide at 12 months at 1 SD below mean depression (low depression), moderate (mean depression) and 1 SD above mean depression (high depression). For mental defeat, measured by PSPS, higher scores are indicative of higher levels of mental defeat. As mental defeat increased the risk of suicide increased, which was significantly moderated by depression.

Simple slopes for the association between mental defeat and risk of suicide risk at 12 months were tested for low (1 SD below the mean), moderate (mean) and high (1 SD above the mean) levels of depression. Each of the simple slope tests revealed a significant positive association between mental defeat and risk of suicide at 12 months, but mental defeat was more strongly related to the risk of suicide at 12 months for high levels of depression (*B* = 0.06, SE = 0.01, *t* = 6.21, *p* < 0.001) than for moderate (*B* = 0.05, SE = 0.01, *t* = 5.20, *p* < 0.001) or lower levels (*B* = 0.04, SE = 0.01, *t* = 2.83, *p* = 0.004) of depression. The moderator value defining Johnson‐Neyman significance regions was −6.56 below the mean (*m* = 7.79, SD = 4.41), representing the region of significant moderation from −6.56 to 13.22 below or above the mean. Figure 2 plots the simple slopes for the interaction.

## 4 Discussion

This study aimed to assess the role of mental defeat in predicting future suicide risk and investigate whether depression moderates the suicide risk conferred by mental defeat. The two main findings of this study are: first, baseline mental defeat and depression are the only variables in our sample predicting the risk of suicide over 12 months in people with chronic pain when considered in a multivariable model; and second, there is a dose–response relationship where depression moderates the relationship between mental defeat and suicide risk at 12 months. Specifically, as mental defeat increased, the risk of suicide increased more strongly for individuals with high levels of depression compared to those with moderate or low levels of depression.

Overall, a strength of our study is that we included a large number of theoretically known predictors of risk of suicide and considered them all (where appropriate) in a single multivariable model. This approach acknowledges the complex nature of symptom presentation. These findings align with previous research, including from short‐term assessments of the present sample, which has shown an association between mental defeat and suicidal thoughts and behaviour alongside other risk factors (Cheatle et al. 2023; Themelis et al. 2023). However, the present study extends these findings by demonstrating the durability of this relationship over a 12‐month timeframe and highlighting the specific roles of mental defeat and depression.

Our previous study had identified additional risk factors for suicide over a six‐month timeframe (Themelis et al. 2023), including perceived stress, pain in the head and active smoking, which were no longer significant when tested over 12 months. A possible explanation could be that factors such as stress and pain in the head may have a more acute effect whereas mental defeat and depression have a more sustained effect. Indeed, the integrated motivational‐volitional model of suicidal behaviour postulates that volitional moderators such as physical pain sensitivity may govern the transition from suicidal ideation to suicidal behaviour (O'Connor 2011; O'Connor and Kirtley 2018). Further research is needed to explore long‐term risk factors (> 1 year) and to understand how they may differ from those that are more relevant in the short term, particularly in chronic pain.

Our findings provide empirical support for underlying prominent theoretical frameworks of suicide. Viewing mental defeat through the lens of theoretical frameworks of suicide can offer insight into potential interactions among various factors or stressors and how they could translate into suicidal behaviour. The Cry of Pain Model (Williams 2001) and the Schematic Appraisal Model (Johnson, Gooding, and Tarrier 2008) feature defeat as a key cognitive factor driving suicidal thoughts and behaviour. The association between mental defeat and risk of suicide in chronic pain has also been conceptualised in the context of the interpersonal‐psychological theory, where feelings of defeat heighten the sense of loss and suffering and motivate sufferers to escape from the self (Tang, Beckwith, and Ashworth 2016). The integrated motivational‐volitional model of suicidal behaviour (O'Connor 2011; O'Connor and Kirtley 2018) proposes a three‐step framework, including a pre‐motivational phase (the context where suicide might occur), the motivational phase (the emergence of suicidal thoughts) and the volitional phase (factors linked to suicide attempts). This model may help explain why depression strengthens the association between mental defeat and suicide risk‐ with depression heightening the threat to the self through prioritising negative self‐referential processing and attention (Beck 1967).

To our knowledge, this is the first prospective study to test whether theory‐derived risk factors predicted and moderated suicide risk in chronic pain over a prolonged period (> 12 months). Our results suggest that mental defeat alongside moderating factors like depression may help explain the variance between mental health conditions and actual suicide attempts (Dhingra, Boduszek, and O'Connor 2015), as well as the variability seen in chronic pain. It would be interesting for future research to examine changes in suicide risk over time and to identify differences in characteristics and circumstances of individuals displaying an increase, no change or a reduction in suicide risk.

Regarding other risk factors, only mental defeat or depression were significant at 12 months when considered in a multivariable model in this sample. This suggests that levels of mental defeat and depression may be more important risk factors than demographical—or pain‐related factors such as pain type or severity. This is in line with previous reports noting inconsistencies in the relationship between pain characteristics and suicide risk in chronic pain (Racine 2018). One possible explanation is that participants in our study reported a wide range of chronic pain conditions with most participants experiencing pain in more than one pain location and high levels of pain severity and pain interference, reflecting its homogenous nature and prevalence in society and therefore helping to produce more relevant predictions.

In this study, we saw that both mental defeat and depression are independent significant predictors of high suicide risk. Consistent with prior research (Tang, Salkovskis, and Hanna 2007; Tang et al. 2010, 2013; Tang, Beckwith, and Ashworth 2016), the findings highlight that mental defeat is not merely a symptom of depression but a construct with its partially separate influence (Themelis et al. 2023). Consequently, efforts targeting mental defeat alongside depression could have important clinical implications for reducing risk of suicide in patients with chronic pain.

### 4.1 Strengths and Limitations

The study's strengths include its prospective design and assessment at different time points, its satisfactory 12‐month retention rate (65.8%), weighted analysis to minimise attrition biases and diverse measurements of primary data within the chronic pain population. However, there are also some limitations to consider. Despite including many variables that are known risk factors of chronic pain, it did not include measures of entrapment or thwarted belongingness due to the lack of validated instruments in the pain population, both of which are key predictors in theories of suicide (O'Connor and Kirtley 2018; Van Orden et al. 2010).

Previous studies have shown a mediating role of entrapment, but not thwarted belongingness in the relationship between defeat and 12‐month suicidal ideation in the general population (Wetherall et al. 2022). Although defeat and entrapment are highly associated constructs in the general population (Forkmann et al. 2018), there has not been much research investigating these constructs in chronic pain, unlike mental defeat.

Though comparable to other longitudinal pain studies, other limitations include a lack of racial/ethnic and educational diversity and exclusion of severe psychiatric comorbidities, potentially underrepresenting the most high‐risk individuals. Replication with more diverse chronic pain groups is crucial and future research should delve into the mechanisms linking mental defeat to suicidal thoughts and behaviours.

## 5 Conclusions

The present study found evidence that, among individuals with chronic pain, those with increased levels of mental defeat are at higher risk of suicide after 12 months and that people with mental defeat are at even higher risk of suicide when they simultaneously experience depression. In the search for additional, specific risk factors for suicide in chronic pain our findings suggest that mental defeat, alongside depression, may offer a novel and valuable target of intervention. This could begin with assessing mental defeat in chronic pain patients presenting with severe depression symptoms. Multiple established cognitive‐behavioural therapy‐based (e.g., image rescripting; cognitive reappraisal and problem‐solving) and acceptance and commitment therapy‐based (e.g., cognitive defusion, flexible perspective taking and compassion) interventions could be adapted to reverse or alleviate mental defeat (Murata et al. 2019; Tang et al. 2020).

## Author Contributions

K.T. and N.K.Y.T. conceived the research idea and developed the theory and plan for the study. K.T. was responsible for setting up the study, data acquisition, formal data analysis and drafting the original manuscript. J.L.G. and P.K. were responsible for data acquisition and management and implementation of governance. K.T. and N.K.Y.T. were responsible for critical revisions of the manuscript. All authors (K.T., J.L.G., P.K., M.D.C., M.A.I., S.B., S.P.S. and N.K.Y.T.) contributed to the study development and reviewed, commented and approved the manuscript.

## Ethics Statement

The authors assert that all procedures contributing to this work comply with the ethical standards of the relevant national and institutional committees on human experimentation and with the Helsinki Declaration of 1975, as revised in 2008.

## Conflicts of Interest

The authors declare no conflicts of interest.

## References

[ejp4779-bib-0049] Aiken, L. S. , and S. G. West . 1991. Multiple Regression: Testing and Interpreting Interactions. Newbury Park, CA: Sage Publications.

[ejp4779-bib-0001] Bair, M. J. , R. L. Robinson , W. Katon , and K. Kroenke . 2003. “Depression and Pain Comorbidity: A Literature Review.” Archives of Internal Medicine 163, no. 20: 2433–2445. 10.1001/archinte.163.20.2433. 14609780

[ejp4779-bib-0002] Bastien, C. H. , A. Vallières , and C. M. Morin . 2001. “Validation of the Insomnia Severity Index as an Outcome Measure for Insomnia Research.” Sleep Medicine 2, no. 4: 297–307. 10.1016/S1389-9457(00)00065-4. 11438246

[ejp4779-bib-0003] Beck, A. T. 1967. Depression: Clinical, Experimental, and Theoretical Aspects. New York: Hoeber Medical Division. Harper & Row. https://worldcat.org/title/2648585.

[ejp4779-bib-0004] Campbell, G. , S. Darke , R. Bruno , and L. Degenhardt . 2015. “The Prevalence and Correlates of Chronic Pain and Suicidality in a Nationally Representative Sample.” Australian and New Zealand Journal of Psychiatry 49, no. 9: 803–811. 10.1177/0004867415569795. 25698809

[ejp4779-bib-0005] Cane, D. , W. R. Nielson , M. McCarthy , and D. Mazmanian . 2013. “Pain‐Related Activity Patterns: Measurement, Interrelationships, and Associations With Psychosocial Functioning.” Clinical Journal of Pain 29, no. 5: 435–442. 10.1097/AJP.0b013e31825e452f. 23247000

[ejp4779-bib-0006] Cheatle, M. D. , N. A. Giordano , K. Themelis , and N. K. Y. Tang . 2023. “Suicidal Thoughts and Behaviors in Patients With Chronic Pain, With and Without Co‐Occurring Opioid Use Disorder.” Pain Medicine 00: 1–8. 10.1093/pm/pnad043. PMC10391589 37014415

[ejp4779-bib-0007] Cheatle, M. D. , T. Wasser , C. Foster , A. Olugbodi , and J. Bryan . 2014. “Prevalence of Suicidal Ideation in Patients With Chronic Non‐cancer Pain Referred to a Behaviorally Based Pain Program.” Pain Physician 17, no. 3: E359–E367. 10.36076/ppj.2014/17/e359. 24850117

[ejp4779-bib-0008] Cheng, H. L. 2016. “A Simple, Easy‐To‐Use Spreadsheet for Automatic Scoring of the International Physical Activity Questionnaire (IPAQ) Short Form.” ResearchGate. Accessed January 6, 2025. 10.13140/RG.2.2.21067.80165.

[ejp4779-bib-0009] Cleeland, C. S. , and K. M. Ryan . 1994. “Pain Assessment: Global Use of the Brief Pain Inventory.” Annals of the Academy of Medicine, Singapore 23, no. 2: 129–138. 8080219

[ejp4779-bib-0010] Cohen, S. , T. Kamarck , and R. Mermelstein . 1983. “A Global Measure of Perceived Stress.” Journal of Health and Social Behavior 24, no. 4: 385–396. 10.2307/2136404. 6668417

[ejp4779-bib-0011] Costanza, A. , V. Chytas , V. Piguet , et al. 2021. “Meaning in Life Among Patients With Chronic Pain and Suicidal Ideation: Mixed Methods Study.” JMIR Formative Research 5, no. 6: e29365. 10.2196/29365. 34003136 PMC8214181

[ejp4779-bib-0012] Craig, C. L. , A. L. Marshall , M. Sjöström , et al. 2003. “International Physical Activity Questionnaire: 12‐Country Reliability and Validity.” Medicine and Science in Sports and Exercise 35, no. 8: 1381–1395. 10.1249/01.MSS.0000078924.61453.FB. 12900694

[ejp4779-bib-0013] Dhingra, K. , D. Boduszek , and R. C. O'Connor . 2015. “Differentiating Suicide Attempters From Suicide Ideators Using the Integrated Motivational‐Volitional Model of Suicidal Behaviour.” Journal of Affective Disorders 186: 211–218. 10.1016/j.jad.2015.07.007. 26247914

[ejp4779-bib-0014] Dueñas, M. , B. Ojeda , A. Salazar , J. A. Mico , and I. Failde . 2016. “A Review of Chronic Pain Impact on Patients, Their Social Environment and the Health Care System.” Journal of Pain Research 9: 457–467. 10.2147/JPR.S105892. 27418853 PMC4935027

[ejp4779-bib-0015] Ehlers, A. , D. M. Clark , E. Dunmore , L. Jaycox , E. Meadows , and E. B. Foa . 1998. “Predicting Response to Exposure Treatment in PTSD: The Role of Mental Defeat and Alienation.” Journal of Traumatic Stress 11, no. 3: 457–471. 10.1023/A:1024448511504. 9690187

[ejp4779-bib-0016] Fairweather‐Schmidt, A. K. , P. J. Batterham , P. Butterworth , and S. Nada‐Raja . 2016. “The Impact of Suicidality on Health‐Related Quality of Life: A Latent Growth Curve Analysis of Community‐Based Data.” Journal of Affective Disorders 203: 14–21. 10.1016/J.JAD.2016.05.067. 27285722

[ejp4779-bib-0017] Fayaz, A. , P. Croft , R. M. Langford , L. J. Donaldson , and G. T. Jones . 2016. “Prevalence of Chronic Pain in the UK: A Systematic Review and meta‐Analysis of Population Studies.” BMJ Open 6, no. 6: e010364. 10.1136/bmjopen-2015-010364. PMC4932255 27324708

[ejp4779-bib-0018] Forkmann, T. , T. Teismann , J. S. Stenzel , H. Glaesmer , and D. De Beurs . 2018. “Defeat and Entrapment: More Than Meets the Eye? Applying Network Analysis to Estimate Dimensions of Highly Correlated Constructs.” BMC Medical Research Methodology 18, no. 1: 16. 10.1186/s12874-018-0470-5. 29370770 PMC5785844

[ejp4779-bib-0019] Gilbert, P. , and S. Allan . 1998. “The Role of Defeat and Entrapment (Arrested Flight) in Depression: An Exploration of an Evolutionary View.” Psychological Medicine 28, no. 3: 585–598. 10.1017/S0033291798006710. 9626715

[ejp4779-bib-0020] Goldberg, D. S. , and S. J. McGee . 2011. “Pain as a Global Public Health Priority.” BMC Public Health 11, no. 1: 770. 10.1186/1471-2458-11-770. 21978149 PMC3201926

[ejp4779-bib-0021] Harden, R. N. , S. R. Weinland , T. A. Remble , et al. 2005. “Medication Quantification Scale Version III: Update in Medication Classes and Revised Detriment Weights by Survey of American Pain Society Physicians.” Journal of Pain 6, no. 6: 364–371. 10.1016/j.jpain.2005.01.350. 15943958

[ejp4779-bib-0048] Hayes, A. F. 2021. Introduction to Mediation, Moderation, and Conditional Process Analysis: A Regression‐Based Approach. 3rd ed. New York: Guilford Publications.

[ejp4779-bib-0022] Johnson, J. , P. Gooding , and N. Tarrier . 2008. “Suicide Risk in Schizophrenia: Explanatory Models and Clinical Implications, the Schematic Appraisal Model of Suicide (SAMS).” Psychology and Psychotherapy: Theory, Research and Practice 81, no. 1: 55–77. 10.1348/147608307X244996. 17919360

[ejp4779-bib-0023] Murata, T. , Y. Hiramatsu , F. Yamada , et al. 2019. “Alterations of Mental Defeat and Cognitive Flexibility During Cognitive Behavioral Therapy in Patients With Major Depressive Disorder: A Single‐Arm Pilot Study.” BMC Research Notes 12, no. 1: 1–7. 10.1186/S13104-019-4758-2/TABLES/3. 31694691 PMC6833291

[ejp4779-bib-0024] Nicholas, M. K. 2007. “The Pain Self‐Efficacy Questionnaire: Taking Pain Into Account.” European Journal of Pain 11, no. 2: 153–163. 10.1016/j.ejpain.2005.12.008. 16446108

[ejp4779-bib-0025] O'Connor, R. C. 2011. “The Integrated Motivational‐Volitional Model of Suicidal Behavior.” Crisis 32, no. 6: 295–298. 10.1027/0227-5910/a000120. 21945841

[ejp4779-bib-0026] O'Connor, R. C. , and O. J. Kirtley . 2018. “The Integrated Motivational‐Volitional Model of Suicidal Behaviour.” Philosophical Transactions of the Royal Society B: Biological Sciences 373, no. 1754: 1–10. 10.1098/rstb.2017.0268. PMC6053985 30012735

[ejp4779-bib-0027] Osman, A. , C. L. Bagge , P. M. Gutierrez , L. C. Konick , B. A. Kopper , and F. X. Barrios . 2001. “The Suicidal Behaviors Questionnaire‐Revised (SBQ‐R): Validation With Clinical and Nonclinical Samples.” Assessment 8, no. 4: 443–454. 10.1177/107319110100800409. 11785588

[ejp4779-bib-0028] Petrosky, E. , R. Harpaz , K. A. Fowler , et al. 2018. “Chronic Pain Among Suicide Decedents, 2003 to 2014: Findings From the National Violent Death Reporting System.” Annals of Internal Medicine 169, no. 7: 448–455. 10.7326/M18-0830. 30208405 PMC6913029

[ejp4779-bib-0029] Racine, M. 2018. “Chronic Pain and Suicide Risk: A Comprehensive Review.” Progress in Neuro‐Psychopharmacology and Biological Psychiatry 87, no. June 2017: 269–280. 10.1016/j.pnpbp.2017.08.020. 28847525

[ejp4779-bib-0030] Roelofs, J. , M. L. Peters , L. McCracken , and J. W. S. Vlaeyen . 2003. “The Pain Vigilance and Awareness Questionnaire (PVAQ): Further Psychometric Evaluation in Fibromyalgia and Other Chronic Pain Syndromes.” Pain 101, no. 3: 299–306. 10.1016/S0304-3959(02)00338-X. 12583873

[ejp4779-bib-0031] Seaman, S. R. , and I. R. White . 2013. “Review of Inverse Probability Weighting for Dealing With Missing Data.” Statistical Methods in Medical Research 22, no. 3: 278–295. 10.1177/0962280210395740. 21220355

[ejp4779-bib-0032] Sullivan, M. J. L. , S. R. Bishop , and J. Pivik . 1995. “The Pain Catastrophizing Scale: Development and Validation.” Psychological Assessment 7, no. 4: 524–532. 10.1037/1040-3590.7.4.524.

[ejp4779-bib-0033] Syrjala, K. L. , A. C. Stover , J. C. Yi , S. B. Artherholt , and J. R. Abrams . 2010. “Measuring Social Activities and Social Function in Long‐Term cancer Survivors Who Received Hematopoietic Stem Cell Transplantation.” Psycho‐Oncology 19, no. 5: 462–471. 10.1002/pon.1572. 19358230 PMC3114555

[ejp4779-bib-0034] Tang, N. K. Y. , P. Beckwith , and P. Ashworth . 2016. “Mental Defeat Is Associated With Suicide Intent in Patients With Chronic Pain.” Clinical Journal of Pain 32, no. 5: 411–419. 10.1097/ajp.0000000000000276. 26201013

[ejp4779-bib-0035] Tang, N. K. Y. , and C. Crane . 2006. “Suicidality in Chronic Pain: A Review of the Prevalence, Risk Factors and Psychological Links.” Psychological Medicine 36, no. 5: 575–586. 10.1017/S0033291705006859. 16420727

[ejp4779-bib-0036] Tang, N. K. Y. , C. E. Goodchild , J. Hester , and P. M. Salkovskis . 2010. “Mental Defeat Is Linked to Interference, Distress and Disability in Chronic Pain.” Pain 149, no. 3: 547–554. 10.1016/j.pain.2010.03.028. 20395047

[ejp4779-bib-0037] Tang, N. K. Y. , C. Moore , H. Parsons , et al. 2020. “Implementing a Hybrid Cognitive‐Behavioural Therapy for Pain‐Related Insomnia in Primary Care: Lessons Learnt From a Mixed‐Methods Feasibility Study.” BMJ Open 10, no. 3: e034764. 10.1136/BMJOPEN-2019-034764. PMC7150590 32193269

[ejp4779-bib-0038] Tang, N. K. Y. , P. M. Salkovskis , and M. Hanna . 2007. “Mental Defeat in Chronic Pain: Initial Exploration of the Concept.” Clinical Journal of Pain 23, no. 3: 222–232. 10.1097/AJP.0b013e31802ec8c6. 17314581

[ejp4779-bib-0039] Tang, N. K. Y. , S.‐H. Shum , P. W. L. Leung , P.‐P. Chen , and P. M. Salkovskis . 2013. “Mental Defeat Predicts Distress and Disability in Hong Kong Chinese With Chronic Pain.” Clinical Journal of Pain 29, no. 9: 830–836. 10.1097/ajp.0b013e3182778153. 23528826

[ejp4779-bib-0040] Themelis, K. , J. L. Gillett , P. Karadag , et al. 2023. “Mental Defeat and Suicidality in Chronic Pain: A Prospective Analysis.” Journal of Pain 24, no. 11: 2079–2092. 10.1016/j.jpain.2023.06.017. 37392929

[ejp4779-bib-0041] Thoemmes, F. , and A. D. Ong . 2016. “A Primer on Inverse Probability of Treatment Weighting and Marginal Structural Models.” Emerging Adulthood 4, no. 1: 40–59. 10.1177/2167696815621645.

[ejp4779-bib-0042] Treede, R. D. , W. Rief , A. Barke , et al. 2019. “Chronic Pain as a Symptom or a Disease: The IASP Classification of Chronic Pain for the International Classification of Diseases (ICD‐11).” Pain 160, no. 1: 19–27. 10.1097/j.pain.0000000000001384. 30586067

[ejp4779-bib-0043] Van Orden, K. A. , T. K. Witte , K. C. Cukrowicz , S. R. Braithwaite , E. A. Selby , and T. E. Joiner . 2010. “The Interpersonal Theory of Suicide.” Psychological Review 117, no. 2: 575–600. 10.1037/A0018697. 20438238 PMC3130348

[ejp4779-bib-0044] Wetherall, K. , S. Cleare , S. Eschle , et al. 2022. “Predicting Suicidal Ideation in a Nationally Representative Sample of Young Adults: A 12‐Month Prospective Study.” Psychological Medicine 52, no. 14: 3168–3175. 10.1017/S0033291720005255. 33634764

[ejp4779-bib-0045] Williams, J. M. G. 2001. The Cry of Pain. London, UK: Penguin Books.

[ejp4779-bib-0046] Woby, S. R. , N. K. Roach , M. Urmston , and P. J. Watson . 2005. “Psychometric Properties of the TSK‐11: A Shortened Version of the Tampa Scale for Kinesiophobia.” Pain 117, no. 1–2: 137–144. 10.1016/j.pain.2005.05.029. 16055269

[ejp4779-bib-0047] Zigmond, A. S. , and R. P. Snaith . 1983. “The Hospital Anxiety and Depression Scale.” Acta Psychiatrica Scandinavica 67, no. 6: 361–370. 10.1111/j.1600-0447.1983.tb09716.x. 6880820

